# On the Extent of the Surface of the Brain, and Its Relations to the Development of the Intellect

**Published:** 1853-04-01

**Authors:** 

**Affiliations:** PHYSICIAN TO THE SALPÊTRIÈRE HOSPITAL


### ON THE EXTENT OF THE SURFACE OF THE BRAIN, AND ITS RELATIONS TO THE DEVELOPMENT OF THE INTELLECT.

BY M. BAILLARGER, PHYSICIAN TO THE SALPÊTRIÈRE HOSPITAL.

(*From the* "*Annales Medico-Psychologiques,*" *for January,* 1853.)

I PROPOSE in this paper,

I. To define the extent of the surface of the cerebral hemispheres.

II. To inquire if there exists, as is said, any relation between the extent of that surface and the degree of intellectual development.

### I.

### *Of the Extent of the Surface of the Brain.*

The brain is covered by two membranes, the arachnoid and the pia-mater.

The arachnoid does not penetrate into the furrows of the convolutions, but simply covers over the outer surface of the cerebral hemispheres.

The pia-mater, on the contrary, dips into all the furrows, so that its surface is precisely equal in extent to the surface of the brain itself. If this membrane could by any manipulation be detached entire and then unfolded, it would afford an easy and exact method of measuring the extent of the cerebral hemispheres; but as this cannot be done, it becomes necessary to resort to some other process.

The first idea which occurs is to unfold the brain itself.

It is known that Gall practised the unfolding of the cerebral hemispheres, and of all his anatomical discoveries it was the one which he regarded as the most important.

Nothing could be more simple than the measurement of the cerebral hemispheres, if it were possible to obtain a plain united surface, in the place of irregularities whose precise extent it is almost impracticable to determine.

Unfortunately, the unfolding of the brain, as practised by Gall, involves a very grave objection.

The cerebral substance is extensible, and the stretching which it undergoes in the manipulation may become a source of error.

This objection ought to stop anatomists from attempting to measure the extent of the surface of the brain by this method, and I have not thought of making use of it.

The process which I employ consists in unfolding the brain, and substituting a long and minute dissection for the use of the fingers, so as to avoid all chances of stretching. I gradually remove as much of the white substance as I can, and so reduce the hemisphere to the thickness of its vesicular layer. When the medullary substance has in this manner been removed as much as is possible, the vesicular portion unfolds of itself, if not entirely, at least sufficiently to enable one to spread it out, and mould it exactly with plaster. Then the cerebral substance is cleared out of the mould, and its surface measured in the following manner:—First, line the inner surface of the plaster cast with some thin and tough membrane; then fill it carefully and entirely with clay; the membrane will thus be forced into all the depressions and irregularities of the mould, and when removed and spread out can be correctly measured.

In fine, to obtain a measurement of the extent of the cerebral hemispheres, one must,

1st. Unfold the hemispheres as much as possible, by gradually removing all the white substance.

2nd. Take a cast in plaster of the vesicular portion so dissected.

3rd. Measure the extent of the internal surface of the cast, by means of a thin membrane carefully applied.

The following are the results which I have obtained by these proceedings:—In five brains I found the mean extent of the entire cerebral surface to be 663 square inches.

In two cases, I measured the comparative extent of surface in the opposite hemispheres, and found in the first brain:

| | sq. in. |
|---|---|
| The surface of the right hemisphere . . . . . . . . . | 297·9* |
| „ left „ . . . . . . . . . | 307·7 |

In the second brain—

| | |
|---|---|
| The surface of the right hemisphere . . . . . . . . . | 332·6 |
| „ left „ . . . . . . . . . | 326·4 |

So that the difference between one hemisphere and the other amounts only to a fiftieth part, which is a proof of the correctness of the measure.

The following are the measurements of the brains of some animals:—

| | sq. in. |
|---|---|
| Surface of a rabbit's brain . . . . . . . . . . . . | 9·36 |
| „ cat's „ . . . . . . . . . . . . | 20·28 |
| „ dog's „ . . . . . . . . . . . . | 40·56 |
| „ sheep's „ . . . . . . . . . . . . | 62·4 |
| „ pig's „ . . . . . . . . . . . . | 95·8 |

Such are the results afforded by my measurings. I do not publish them as mathematically correct, but I venture to guarantee their exactness within a fifteenth part.

### II.

### *Of the Relation between the Extent of the Surface of the Brain and the Degree of Intellectual Development.*

Desmoulins, in 1822, attempted to demonstrate, that the number and perfection of the intellectual faculties, in the different species of animals, and in different individuals of the same species, were proportionate to the extent of the cerebral surface.

I do not think it necessary to repeat all the arguments which were brought forward to support this proposition, and I will make but two remarks.

It is said that an intelligent brute has a much larger extent of cerebral surface than a brute less intelligent. Of course this means extent of surface in relation to the total bulk; for, taken absolutely, the brain of a dog has a smaller surface than the brain of an ox, yet the dog is much the more intelligent. In a comparison, therefore, of the extent of surface, the relative total bulk must be taken into account. It is thus, probably, that these comparisons have been made; nevertheless, it would have been as well if this had been more clearly stated.

* We have reduced the French measures to English.—TR.

I will add a second observation. Desmoulins, and the physiologists who have supported his opinions, have never given any measurements, even approximative, of the cerebral surfaces.

Nevertheless in a question of this kind, especially when an attempt is made to estimate the comparative development of the intelligence, resort should have been had to the scales and measure.

It is indispensable, therefore, in attempting the solution of this problem, first, to ascertain the exact volume of each brain; secondly, to measure the extent of its surface. Having carefully weighed the human brain, then that of the pig, sheep, dog, cat, and rabbit, I next proceeded to measure their respective surfaces. To facilitate the comprehension of the results, I will first take the two extremes, the human brain and that of the rabbit.

| | |
|---|---|
| I found that the cerebral hemisphere of man, after the removal of the membranes, the corpora striata, the optic thalami, and the corpus callosum, weighed . . . . . . . . . . | Troy. lbs. oz. dwts. 2 4 18 |
| Their surface measured by the method above described, was . | 663 sq. in. |
| The cerebral hemisphere of a rabbit weighed . . . . . . | 3 dwts. 5 grs. troy. |
| Their surface is equal to . . . . . . . . . . . . . | 9·36 sq. in. |

On comparing the weights of the two brains, it is seen that the human brain weighs 180 times more than the brain of a rabbit, so that the ratio is as 1 to 180; whilst the human brain has an extent of surface 70 times greater than that of the rabbit, being a ratio of 1 to 70.

Thus, although the human brain weighs 180 times as much as that of a rabbit, it has only 70 times the extent of surface; and the cerebral hemispheres of a rabbit have, relatively to their weight, or otherwise to their volume, a surface two and a half times greater than that of man.

I ought perhaps to remind the reader, that the rabbit's brain, like all the inferior mammals, is destitute of convolutions; so that its external surface is an exact measure of the internal capacity of the cranium.

I have compared, in the same manner, the human brain with that of the cat, dog, sheep, and hog, and I have always found that the extent of surface is, relatively to the weight, a quarter or a third larger in animals than in man. The smallest brains have comparatively the largest extent of surface, so that the amount of surface seems in inverse proportion to the weight.

These results are diametrically opposed to those stated by Desmoulins; for so far from the amount of intelligence being in direct proportion to the extent of the cerebral surfaces, I have found it precisely the reverse.

I at first mistrusted these facts, which I established without understanding them; but having sought an explanation in mathematical science, I found the following:—Suppose two spherical bodies of the same density, one 20 inches, the other 10 inches in diameter. The volumes, which are proportionals of the weights, are as 8 to 1—that is to say, the larger of the bodies is 8 times heavier than the smaller. But when the surfaces are compared, they are found to be as 4 to 1. Thus the larger body is 8 times heavier than the smaller, but only 4 times as large.

This is precisely what I found on comparing the surface of the brains of animals with that of man.

These differences between the relative volumes and surfaces result from the mathematical laws, that the volumes of similar bodies are proportionate to the cubes of their diameters; whilst their surfaces are proportionate to the squares of their diameters.

The brain is subject to this law, although allowance must be made for the convolutions. This is the reason why the cerebral hemispheres of the rabbit are, notwithstanding the absence of convolutions, nearly three times larger, proportionably, than those of man. There is, therefore, nothing extraordinary in the results which I obtained from the measurements of the surfaces of the human brain and those of animals. However, they completely upset the opinion, that the number and perfection of the intellectual faculties are proportionate to the extent of the cerebral surfaces.

I have stated, that the brain is only in part subject to the mathematical law which regulates the proportions between the bulk and the surfaces of regular spherical bodies; but there was nothing to prevent its being entirely removed from this law. I cannot better demonstrate this than by recalling the extent of the surface of the cerebellum as compared with its weight. This extent, when the numberless convolutions are included, is considerable, relative to the bulk, and renders it the only part of the human brain which, in this respect, can be compared with the brains of the inferior mammals.

The same proportions might have obtained in the case of the cerebral hemispheres, and then perhaps the degree of intellectual development would have been proportionate to the extent of the cerebral surfaces, which is not the fact.

I think it right, before I conclude, to point out the source of the error committed by Desmoulins and the physiologists who have supported his opinions.

The relative extent of the cerebral surface has been estimated by the foldings it presents, or, in other words, by the number and projection of the convolutions; but this is not exact.

The human brain, which has numerous strongly-marked convolutions, has relatively but a small extent of surface, in proportion to its great bulk.

In solving this problem, the following points should have been considered:—

1st. The relative extent of surface in different brains.

2nd. The number and comparative development of the convolutions.

I believe I have proved that the perfection of the intelligenee is not directly proportionate to the extent of the cerebral surfaces; yet it remains to be examined whether some relation of the kind may not be established, by taking into account, not the extent of surface, but the number and depth of the convolutions. For, changing the terms of the proposition, it may be stated, that the amount and perfection of the intellectual faculty is proportionate to the number and depth of the convolutions; which is henceforth the question to determine.

The solution of this question, it appears to me, presents much greater difficulties than have been supposed to belong to it. In fact, it is not sufficient to compare the external appearance of two brains of different animals, and to state that the brain of the more intelligent brute presents the greater number of convolutions. The problem is much more complicated, and its satisfactory solution involves several important elements which have hitherto been neglected.

### CONCLUSIONS.

1. The human brain may be unfolded, almost entirely without stretching, by gradually removing the internal white structure.
2. The extent of the vesicular layer of the cerebral hemispheres thus unfolded, is about 663 square inches.
3. The surface of the human brain, proportionably to its bulk, is much less than that of the inferior mammals.
4. It is impossible to estimate correctly the relative extent of surface in various brains of different volumes, simply by taking account of the number and depth of their convolutions.
5. The degree of intellectual development is not in direct proportion to the extent of the cerebral surfaces, but rather in the inverse ratio.*

* I think it necessary to repeat, that this does not prove that the development of the intellect is not proportionate to the number and depth of the convolutions; for, on reflection, it will be apparent that there is no contradiction in these two propositions.

